# Comparing health-related quality of life of Dutch and Chinese patients with traumatic brain injury: do cultural differences play a role?

**DOI:** 10.1186/s12955-017-0641-9

**Published:** 2017-04-14

**Authors:** Maryse C. Cnossen, Suzanne Polinder, Pieter E. Vos, Hester F. Lingsma, Ewout W. Steyerberg, Yanming Sun, Pengpeng Ye, Leilei Duan, Juanita A. Haagsma

**Affiliations:** 1 Center for Medical Decision Sciences, Department of Public Health, Erasmus Medical Center, P.O. Box 2040, 3000 CA Rotterdam, The Netherlands; 2 Department of Neurology, Slingeland Hospital, Doetinchem, The Netherlands; 3 Beijing Centers for Disease Control and Prevention, Beijng, People’s Republic of China; 4 NCDC, China CDC, Beijing, People’s Republic of China; 5 Institute for Health Metrics and Evaluation, University of Washington, Seattle, USA

**Keywords:** Quality of life, SF-36, Traumatic brain injury, Cultural comparison, Health domains

## Abstract

**Background:**

There is growing interest in health related quality of life (HRQoL) as an outcome measure in international trials. However, there might be differences in the conceptualization of HRQoL across different socio-cultural groups. The objectives of current study were: (I) to compare HRQoL, measured with the short form (SF)-36 of Dutch and Chinese traumatic brain injury (TBI) patients 1 year after injury and; (II) to assess whether differences in SF-36 profiles could be explained by cultural differences in HRQoL conceptualization. TBI patients are of particular interest because this is an important cause of diverse impairments and disabilities in functional, physical, emotional, cognitive, and social domains that may drastically reduce HRQoL.

**Methods:**

A prospective cohort study on adult TBI patients in the Netherlands (RUBICS) and a retrospective cohort study in China were used to compare HRQoL 1 year post-injury. Differences on subscales were assessed with the Mann-Whitney *U*-test. The internal consistency, interscale correlations, item-internal consistency and item-discriminate validity of Dutch and Chinese SF-36 profiles were examined. Confirmatory factor analysis was performed to assess whether Dutch and Chinese data fitted the SF-36 two factor-model (physical and mental construct).

**Results:**

Four hundred forty seven Dutch and 173 Chinese TBI patients were included. Dutch patients obtained significantly higher scores on role limitations due to emotional problems (*p* < .001) and general health (*p* < .001), while Chinese patients obtained significantly higher scores on physical functioning (*p* < .001) and bodily pain (*p* = .001). Scores on these subscales were not explained by cultural differences in conceptualization, since item- and scale statistics were all sufficient. However, differences among Dutch and Chinese patients were found in the conceptualization of the domains vitality, mental health and social functioning.

**Conclusions:**

One year after TBI, Dutch and Chinese patients reported a different pattern of HRQoL. Further, there might be cultural differences in the conceptualization of some of the SF-36 subscales, which has implications for outcome evaluation in multi-national trials.

**Electronic supplementary material:**

The online version of this article (doi:10.1186/s12955-017-0641-9) contains supplementary material, which is available to authorized users.

## Background

Health-related quality of life (HRQoL) reflects an individual’s perception of how an illness and its treatment affect physical, mental and social aspects of his/her life [1]. Because it provides well-standardized information on recovery patterns, frequency, nature, and predictors of disabilities, HRQoL has been recognized as an important outcome in many medical fields, including injury [2]. Similarly, there is growing interest in international HRQoL assessment as a result of the increasing number of international trials [3].

Traumatic brain injury (TBI) is a major public health concern with a rising incidence all over the globe. In Europe, the annual number of hospital admissions is estimated at 262 per 100,000 population [4]. In other parts of the world, data on TBI incidence is less often collected systematically. Nevertheless, a 2004 epidemiological study in Eastern China found that the incidence of TBI among 77 hospitals was substantial [5]. TBI is an important cause of impairments and disability in functional, physical, emotional, cognitive, and social domains that may drastically reduce HRQoL [6, 7]. As a consequence, HRQoL has been emerged as an important outcome measurement following TBI [8].

Previous literature has indicated that there might be differences in the experience and conceptualization of HRQoL across different socio-cultural groups [9–14]. For example, in Western countries body and mind are usually regarded as two different entities, whereas Asian cultures have a more holistic sense among body and mind [15]. Therefore, the strict dichotomization of physical versus mental health, which is often included in HRQoL assessment, might not be applicable to Asian cultures [9, 12]. Also, previous evaluations of the short form (SF)-36 among Asians have shown that they conceptualize social role functioning differently from Western populations [9, 10, 12, 16, 17]. For example, Asians are more directed towards others and the use of “sickness” as an excuse for avoiding social and labour responsibilities is considered unacceptable in the Asian culture [10, 15]. Furthermore, while Western populations associate energy level strongly with physical health, Asians associate energy more strongly with mental health [10–13, 18].

To our knowledge, there is no previous study that directly compared HRQoL between Western and Asian patients after injury. The purpose of this study was to compare HRQoL, measured with the SF-36, of Dutch and Chinese TBI patients 1 year after the injury. Secondly, we aimed to assess whether potential differences in SF-36 profiles between these patients could be explained by cultural differences in HRQoL conceptualization.

## Methods

This study was conducted and reported according to the ‘Strengthening the Reporting of Observational Based Studies’ (STROBE) statement version 4 [19].

### Participants

Data for the current study were obtained from two cohort studies performed in the Netherlands and China. The Radboud University Brain Injury Cohort Study (RUBICS) includes patients aged 16 years and older with mild, moderate and severe TBI presenting at the emergency department (ED) of a level I trauma center in Nijmegen, the Netherlands. Patient demographics, clinical characteristics as well as outcome measurements after 12 months follow-up were prospectively collected between June 2003 and June 2010. More information on data collection and included patients can be found in previous publications [20–24]. Data on Chinese patients were obtained from a retrospective study on injury patients admitted to one of three national injury surveillance hospitals in Zhuhai, Guangdong Province, China between January and December 2006. Patients were 15 years or older and were examined at 12 months post-injury. Data on age, gender and injury severity were collected from the hospital database. No other baseline and injury characteristics that might be relevant in the current study (e.g., education, Glasgow Coma Scale) were measured. More information about this study can be found in a previous publication [25].

To warrant comparability of patient groups, the following inclusion criteria to determine eligibility for current study were used: age ≥16 years, admitted to the hospital with a clinical diagnosis of TBI, provision of informed consent and completion of at least all items of one SF-36 subscale after 12 months follow-up. Patients referred home after the ED visit and patients who died within the first year post-injury were excluded.

### TBI definition and classification

In the Dutch dataset, all patients sustained a TBI. Consequently, all patients meeting the inclusion criteria for the current study were included in the analyses. The Chinese dataset was not restricted to patients with TBI, but contained patients with various injuries. The TBI patients were selected by including all patients with an International Classification of Diseases and Related Health Problems (ICD-10) code of S06, referring to traumatic intracranial injury.

Severity of TBI was determined by the Abbreviated Injury Scale - Head (AISH). The AISH is, together with the Glasgow Coma Scale (GCS), the most commonly used index of severity in TBI [26]. Severity of TBI is ranked on a scale from 1 to 6 in which 1 being mild, 2 being moderate and 6 being unsurvivable [27]. Patients were classified into mild/moderate and severe TBI according to their AISH score (1–2 versus >2).

The Chinese dataset did not report data on AISH. However, ICD-10 codes can be translated into AISH scores by using the ICD/AIS MAP [28, 29]. Consequently, those patients with ICD-10 codes of S06.0, S06.1, S06.2 and S06.9 were classified as having mild or moderate TBI and those with ICD-10 codes of S06.3, S06.4, S06.5, S06.6, S06.7 and S06.8 were classified as having severe TBI.

### Measurement of HRQoL

The SF-36 was used to measure 12-month HRQoL. The SF-36 is the most frequently used generic instrument for HRQoL [30] and has adequate internal consistency and validity in TBI patients [31, 32]. The questionnaire has been translated and tested in more than 50 languages [30], including Dutch [33] and Cantonese [30]. The SF-36 has two versions (version 1 and version 2) that differ slightly in wording, lay-out and the fact that the role questions have a dichotomous answer category in version 1 and a 5-point scale in version 2.

The SF-36 yields a profile of the following eight concepts: physical functioning (PF), role limitations related to physical health problems (RP), bodily pain (BP), general health perceptions (GH), vitality (VT), social role functioning (SF), role limitations related to emotional health problems (RE) and mental health (MH). The raw scores for each concept were transformed into a 0–100 scale in which higher scores indicated better HRQoL.

In the Dutch dataset, the 12-month SF-36 version 1 was administered by a postal questionnaire that was sent to all patients. In the Chinese dataset, the 12-month SF-36 version 1 was administered by a telephone interview. Patients were interviewed by a hospital nurse who received specific interview training [25].

### Statistical analyses

Differences between patients included in the study and those lost to follow-up were calculated using the non-parametric Mann-Whitney *U* test for continuous data and Chi square test for categorical data. Similarly, Dutch and Chinese patients included in this study were compared using these statistical tests on age, gender and TBI severity.

Means, standard deviations, medians, interquartile ranges and the percentage of patients with the highest (“ceiling”) and lowest (“floor”) scores on the SF-36 subscales were calculated for Dutch and Chinese patients classified by TBI severity. Since the number of severe TBI patients in the Chinese dataset was small (*n* = 20), the analyses were continued with mild and moderate TBI only.

Differences in SF-36 subscales between the Dutch and Chinese patients were calculated with the Mann-Whitney *U* test, since all subscales had a skewed distribution. To allow for multiple testing, a stringent *p*-value of 0.0065 (0.05 divided by 8 subscales) was considered statistically significant. To assess whether differences between Dutch and Chinese patients could be explained by age differences between both populations, the sample was stratified into three equal age groups based on percentiles (33th and 66th) in the total population and the analyses were repeated accordingly. Since sample sizes of the age cohorts were small, statistically significance was assessed on both the stringent *p*-value (*p* < .0065) and the standard *p*-value (*p* < .05).

To examine whether there were differences in cultural conceptualization of HRQoL among Dutch and Chinese patients, the psychometric assumptions underlying the construction of the SF-36 were assessed for both Dutch and Chinese patients. Therefore, the reliability coefficient (“Cronbach’s alpha”) for each subscale was estimated. Adequate internal consistency was defined as a reliability coefficient ≥ 0.70 [34]. Additionally, the reliability coefficient of each subscale should be larger than the subscale’s interscale correlations with all other subscales [35].

Item-internal consistency and item-discriminate validity of the 35 items in both datasets were subsequently assessed. One item (“health change”) was excluded since this provides an indication of perceived change in health rather than the health status 1 year post-injury. The correlation between each item and its hypothesized subscale (“corrected item-to-scale correlation”) should be at least 0.40 for adequate item-internal consistency [35, 36]. Item-discriminate validity was considered adequate if the correlation between an item and its hypothesized scale was larger than the correlations between that item and all other subscales [35].

To examine whether Dutch and Chinese SF-36 subscales reflected the same underlying dimensions, i.e., a physical and mental dimension [30, 37], confirmatory factor analysis (CFA) with two latent constructs was performed. Based on theory and research in Western populations, it was hypothesized that the PF, RP and BP subscales were associated with the physical construct, whereas the MH, RE and SF subscales were associated with the mental construct [30, 37]. For VT and GH it was expected that they load equally on both components [30, 37]. To achieve model identification, for every latent variable one factor loading was fixed to one (PH for physical construct; MH for mental construct; Additional file 1). Maximum likelihood methods were used to estimate the associations between subscales and latent factors. The Tucker-Lewis Index (TLI; recommended > 0.95), Comparative Fit Index (CFI; recommended > 0.95) and the Root mean Square Error of Approximation (RMSE; recommended < 0.08) were used to examine model fit, as recommended by previous research [38]. The CFA analyses were performed using the Analysis of Moment Structures (AMOS) version 4 statistical software package. All other analyses were performed using Statistical Package for the Social Sciences (SPSS) version 21.

## Results

### Study population

The Dutch dataset consists of 2286 TBI patients. Of these patients, 223 were excluded because they were younger than 16 years and 804 patients were subsequently excluded because they did not receive the follow-up questionnaires because of various reasons (e.g., dementia, unknown address). Three hundred sixty patients were further excluded because they were not admitted to the hospital after the ED visit. This results in 899 eligible patients of whom 447 completed all items of at least one of the SF-36 subscales after 12-month follow-up. Patients with a missing 12-month SF-36 did not differ from those included in this study on age and gender. Those lost to follow-up were however less often diagnosed with severe TBI (*p* < .01). Of the included patients, 64% was male and the median age was 46 years (interquartile range 27–58). Half of the patients had an AISH of 1–2, indicating mild and moderate TBI.

The Chinese dataset comprises information on 3664 injury patients of whom 695 patients were diagnosed with TBI according to their ICD-10 codes. Fourty-five patients were removed since they were younger than 16 years of age. Of the 650 eligible patients, 173 (27%) completed the 12 month follow-up assessment. The main reason for non-inclusion in the study was that the telephone number was not available in the hospital database [25]. Respondents were significantly older (median age respondents = 36; median age non-respondents = 32, *p* = .01) and less often diagnosed with severe TBI (respondents: 12% severe TBI, non-respondents: 18% severe TBI, *p* = 0.04). Median age of the included patients (*n* = 173) was 35 years (interquartile range 24–50) and 67% of the study population was male. The large majority (88%) had an AISH of 1 or 2 (mild or moderate TBI).

Dutch and Chinese patients did not differ in terms of gender. Dutch patients were however significantly older than Chinese patients (*p* < .001) and were significantly more often diagnosed with severe TBI (*p* < .001). Comparison of other demographic and clinical characteristics between patient groups was not possible since these were not measured in the Chinese data.

### SF-36 scores of Dutch and Chinese patients

Scores on SF-36 subscales for Dutch and Chinese patients, stratified by TBI severity, are presented in Table 1. Generally, severe TBI patients seemed to report more problems with HRQoL than mild and moderate TBI patients. Ceiling effects were prominent for both Dutch and Chinese patients; more than half of the patients obtained a maximum score for role limitations due to physical problems. In the Dutch dataset, the strong ceiling effect was also shown for role limitations due to emotional problems, while in the Chinese dataset more than half of the patients obtained a maximum score for physical functioning. Since the Chinese dataset included 20 patients with severe TBI, all subsequent analyses were performed for only those patients with mild and moderate TBI.

**Table 1:** Short Form (SF)-36 scores of Dutch and Chinese traumatic brain injury patients 12 months post-injury

Nijmegen, the Netherlands												
	Abbreviated Injury Score Head 1–2						Abbreviated Injury Score Head >2					
	N	Range	Mean (SD)	Median (IQR)	Floor (%)^a^	Ceiling^a^ (%)	N	Range	Mean (SD)	Median (IQR)	Floor (%)^a^	Ceiling (%)^a^
PF	200	0–100	81.2 (24.6)	95 (70–100)	0.5%	38.0%	207	0–100	77.5 (28.5)	90 (70–100)	4.8%	30.0%
RP	211	0–100	68.1 (40.7)	100 (25–100)	19.9%	55.5%	214	0–100	56.7 (43.9)	75 (0–100)	29.9%	44.4%
BP	217	0–100	73.9 (26.2)	80 (52–100)	0.9%	38.2%	216	0–100	75.9 (25.2)	82 (62–100)	1.4%	40.7%
GH	213	0–100	68.5 (22.8)	72 (52–87)	0.5%	3.8%	216	10–100	70.2 (20.0)	72 (60–87)	0%	4.2%
VT	215	5–100	65.0 (21.3)	65 (50–80)	0%	5.6%	218	5–100	64.6 (20.3)	65 (50–80)	0%	4.6%
SF	217	13–100	81.5 (22.2)	88 (63–100)	0%	44.2%	220	13–100	78.3 (22.5)	88 (63–100)	0%	37.3%
RE	214	0–100	81.5 (34.2)	100 (67–100)	11.7%	72.9%	217	0–100	75.6 (38.1)	100 (67–100)	16.1%	65.9%
MH	216	0–100	74.5 (20.2)	80 (64–88)	0.5%	6.9%	218	20–100	73.8 (20.1)	80 (63–88)	0%	6.0%
Zhuhai, China												
	Abbreviated Injury Score Head 1–2						Abbreviated Injury Score Head > 2					
	N	Range	Mean (SD)	Median (IQR)	Floor (%)^a^	Ceiling (%)^a^	N	Range	Mean (SD)	Median (IQR)	Floor (%)^a^	Ceiling (%)^a^
PF	153	5–100	93.0 (16.8)	100 (95–100)	0%	64.7%	20	0–100	82.3 (26.2)	95 (75–100)	5.0%	45%
RP	153	0–100	68.8 (40.6)	100 (25–100)	19.6%	56.2%	20	0–100	60.0 (44.7)	88 (6–100)	25.0%	50%
BP	153	0–100	81.7 (26.4)	100 (67–100)	1.3%	56.9%	20	10–100	70.8 (27.8)	79 (52–97)	0%	25%
GH	153	5–100	58.0 (23.9)	60 (40–75)	0%	2.6%	20	15–85	51.5 (19.5)	53 (40–65)	0%	0%
VT	153	0–100	66.6 (23.8)	70 (50–85)	1.3%	7.8%	20	15–100	67.5 (24.8)	78 (46–85)	0%	10%
SF	153	11–100	85.0 (21.7)	89 (78–100)	0%	49.7%	20	33–100	80.6 (23.9)	89 (58–100)	0%	45%
RE	153	0–100	55.3 (41.0)	67 (0–100)	26.8%	37.3%	20	0–100	60.0 (44.1)	67 (0–100)	30.0%	45%
MH	153	4–100	75.6 (20.4)	80 (64–90)	0%	11.8%	20	48–100	81.0 (14.0)	82 (72–92)	0%	10%

*Note:* Scale scores range from 0 to 100, with 100 representing optimal functioning

*Abbreviations*: *SD* Standard deviation, *IQR* Interquartile range, *PF* Physical functioning, *RP* Role physical, *BP* Bodily pain, *GH* General health, *VT* Vitality, *SF* Social functioning, *RE* Role-emotional, *MH* Mental health

^a^Floor (%) refers to the percentage of patients with the lowest score on a subscale (score 0); Ceiling (%) refers to the percentage of patients with the highest score on a subscale (score 100)

**Fig. 1:**
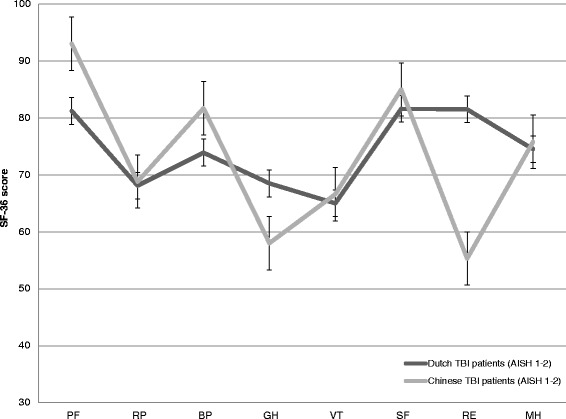
Short Form (SF)-36 score profiles of Dutch and Chinese patients. *Note.* Figure shows SF-36 score profiles of Dutch and Chinese patients with mild and moderate traumatic brain injury 12 months post-injury. Scale scores range from 0 to 100, with 100 representing optimal functioning. Abbreviations*.* PF = physical functioning; RP = role physical; BP = bodily pain; GH = general health; VT = vitality; SF = social functioning; RE = role-emotional; MH = mental health; AISH = Abbreviated Injury Scale Head

When using the stringent *p*-value (*p* < .0065), Chinese patients obtained significantly higher scores on the subscales PF (*p* < .001) and BP (*p* = .001), while Dutch patients obtained higher scores on RE (*p* < .001) and GH (*p* < .001; see Fig. 1 and Additional file 2). Chinese patients also obtained higher scores on SF (*p* = .026), but this was not statistically significant using the stringent *p*-value.

Age differences between Dutch and Chinese patients did not explain the differences in the PF and RE scale scores, since differences remained statistically significant in the different age cohorts (*p* <. 0065 in two age cohorts; *p* < .05 in one age cohort, see Additional file 2). For BP, however, the statistically significant differences between Dutch and Chinese patients did not withstand after stratification for age (no significant differences between Dutch and Chinese patients in 2 out of 3 age strata, see Additional file 2). With regard to GH, Dutch patients obtained significantly higher scores in two out of three age cohorts (*p* < .0065). In the youngest age cohort, however, no statistically significant differences were found between Dutch and Chinese patients.

### Cultural conceptualization of HRQoL

In the Dutch dataset, all SF-36 subscales had an adequate internal consistency and none of the intercorrelations between subscales were larger than the values of Cronbach’s alpha (see Table 2). Item-internal consistency and item-discriminate validity were also adequate for all items. One of the items of the vitality scale (VT1), nevertheless, correlated higher with the MH scale (r = 0.55) than with the VT scale itself (r = 0.49; see Additional file 3).

**Table 2:** Reliability coefficients (in diagonals) and Pearson’s correlation coefficients between Short Form (SF)-36 subscales in patients with mild and moderate traumatic brain injury

Nijmegen, the Netherlands									Zhuhai, China								
	PF	RP	BP	GH	VT	SF	RE	MH		PF	RP	BP	GH	VT	SF	RE	MH
PF	(.94)								PF	(.93)							
RP	.73**	(.90)							RP	.53**	(.90)						
BP	.64**	.71**	(.88)						BP	.47**	.68**	(.90)					
GH	.54**	.68**	.56**	(.83)					GH	.44**	.69**	.61**	(.76)				
VT	.41**	.60**	.50**	.66**	(.75)				VT	.30**	.50**	.43**	.51**	(.66)			
SF	.54**	.67**	.54**	.63**	.73**	(.83)			SF	.53**	.64**	.61**	.59**	.50**	(.49)		
RE	.38**	.59**	.37**	.47**	.55**	.66**	(.86)		RE	.26**	.56**	.41**	.49**	.47**	.45**	(.78)	
MH	.31**	.50**	.35**	.61**	.78**	.72**	.61**	(.89)	MH	.28**	.47**	.40**	.47**	.63**	.51**	.47**	(.70)

*Note.* Table shows reliability coefficients and Pearson’s correlation coefficients between SF-36 subscales in patients with mild and moderate traumatic brain injury 12 months post-injury

*Abbreviations: PF* Physical functioning, *RP* Role physical, *BP* Bodily pain, *GH* General health, *VT* Vitality, *SF* Social functioning, *RE* Role-emotional, *MH* Mental health

In the Chinese dataset, internal consistency was insufficient for two subscales (VT and SF). Also, the intercorrelations between SF and six other subscales were larger than the value of Cronbach’s alpha for the SF scale. Item-internal consistency and item-discriminate validity were adequate for the large majority of items. However, four items (VT2, SF1, SF2 and MH3) obtained a corrected item-to-scale correlation below 0.40. Furthermore, some items from the GH, VT, SF and MH subscales correlated higher with other subscales than with their own hypothesized subscales (see Additional file 3).

CFA with a two-factor model in the Dutch population resulted in a TLI of 0.88, a CFI of 0.95 and an RMSEA of 0.13, indicating a mixed pattern of model fit. The associations between the SF-36 subscales and the two latent constructs was as hypothesized for seven subscales (Table 3). The VT subscale, however, was strongly associated with the mental component (ß = 1.08, *p* < .01) but not with the physical component (ß = 0.01, *p* = 0.94). The association between the physical and mental health construct was strong in the Dutch data (*r* = 0.70).

**Table 3:** Confirmatory Factor Analysis of the Short Form (SF)-36 subscales

		Nijmegen, the Netherlands			Zhuhai, China		
Observed variable	Latent construct	ß	B	*p*-value	ß	B	*p*-value
PF	Physical	1.00^a^	0.78	NA	1.00^a^	0.60	NA
RP	Physical	2.02	0.94	< .01	3.58	0.88	< .01
BP	Physical	1.04	0.76	< .01	2.04	0.77	< .01
GH	Physical	0.48	0.45	< .01	1.25	0.52	.01
VT	Physical	0.01	0.01	.94	−2.31	−0.97	.18
GH	Mental	0.59	0.45	< .01	0.49	0.28	.14
VT	Mental	1.06	0.86	< .01	2.87	1.65	.03
SF	Mental	1.13	0.88	< .01	1.19	0.75	< .01
RE	Mental	1.39	0.70	< .01	1.89	0.63	< .01
MH	Mental	1.00^a^	0.86	NA	1.00^a^	0.67	NA

*Note:* Table represents unstandardized (ß) and standardized (B) regression weights between subscales and the physical and mental component for both the Dutch and the Chinese mild and moderate traumatic brain injury patients 12 months post-injury

*Abbreviations: PF* Physical functioning, *RP* Role physical, *BP* Bodily pain, *GH* General health, *VT* Vitality, *SF* Social functioning, *RE* Role-emotional, *MH* Mental health

^a^Regression weight was set to 1.00

*Statistically significant (*p* < .05) association

● = strong association between subscale and rotated principal component is expected based on previous research [30, 37]

◌ = weak/no association between subscale and rotated principal component is expected based on previous research [30, 37]

CFA with a two-factor model in the Chinese population had an adequate model fit (TLI: 0.95, CFI: 0.97 and RMSE: 0.08). However, the VT scale was negatively associated with the physical construct (ß = −2.31, *p* = .18) and the association between the mental construct and VT (ß = 2.87) was larger than its correlation with MH (ß = 1.00; Table 3). In addition, the association between GH and the physical construct (ß = 1.25, *p* = .01) was larger than the association between GH and the mental construct (ß = 0.49, *p* = .14). The correlation between the physical and mental health construct was very strong (*r* = 0.92) in the Chinese data.

## Discussion

Dutch and Chinese patients with mild and moderate TBI showed a different HRQoL pattern 1 year post-injury. Dutch patients reported less role limitations due to emotional problems and a better general health, whereas Chinese patients reported better physical functioning and less bodily pain. Differences in these subscales cannot be explained by variation in cultural conceptualization. However, there were differences in the conceptualization of some of the other subscales (vitality, mental health and social functioning).

Differences in SF-36 profiles among Dutch and Chinese patients were also recently found in cardiac patients [39, 40]. There are various hypotheses that may explain these differences. Firstly, Dutch and Chinese patients might value similar symptoms and limitations differently. In China, health is usually described as a balance between “yin and yang” and the appreciation of one’s health is largely influenced by spirituality [15]. In the Dutch culture, on the opposite, HRQoL might be more related to the number and severity of symptoms. In addition, because cultural values emphasize harmony in Asian cultures, Asians might be more optimistic when experiencing similar symptoms and less likely to report negative and extreme feelings [41]. Related, coping strategies of Dutch and Chinese patients might vary, since these are largely influenced by cultural systems [9]. Another hypothesis might be that the differences between Dutch and Chinese patients, especially in the physical health dimension, reflect the variation in acute and rehabilitation treatment between countries. In China, a part of the TBI related care is not reimbursed [42] and therefore, it is possible that some of the Chinese patients included in this study did not receive adequate acute or rehabilitative care, influencing their HRQoL 1 year post-injury. Lastly, the differences between Dutch and Chinese patients might also be explained by a lack of comparability of the included patients (e.g., there might have been baseline differences between patients) and study designs (prospective study with postal questionnaire versus retrospective study with telephone interview).

Our finding that social functioning is conceptualized differently among Dutch and Chinese mild and moderate TBI patients is consistent with previous research about psychometrics of the SF-36 in Asian cultures [9, 10, 14, 16–18]. It has been suggested that the concept of social functioning is more Westernized and less clear for Asian people [14]. The strong association between vitality and mental health in our Chinese sample was also consistent with previous literature of the general population [11, 13, 14, 18]. In traditional Chinese medicine a mental disorder is referred to as “the loss of a vital substance of spirit” [17], which could explain this strong association. Notwithstanding, we also found that vitality was strongly associated with mental health but not with physical health in the Dutch population, suggesting that this association could also be related to the TBI rather than to cultural conceptualization. The sequelae of mild and moderate TBI often includes mental health problems as well as fatigue or lack of energy [43, 44], whereas physical problems, such as headache, usually resolve within a few months [45]. Since this is the first study that performed CFA with the SF-36 in a TBI population, current findings should be confirmed by future studies with larger numbers of patients. The high correlation between mental and physical health in Chinese patients may indicate that these patients have a more holistic sense among body and mind [15]. As a consequence, one latent factor rather than two (physical and mental health) might have been more appropriate for the Chinese patients. This should also be confirmed in studies with larger sample sizes.

This is the first study that directly compared HRQoL between Asian and Western patients after injury. A strength of current study is that we did not only assess differences on the SF-36 subscales between Dutch and Chinese patients, but also examined whether these differences could be explained by cultural differences in the conceptualization of quality of life. In addition, we stratified our analyses for age and severity and included an adequate sample size.

Results should however be interpreted in the light of the following limitations. First, response rates were relatively low (50% for the Netherlands and 27% for China) for both datasets. Although low response rates do not necessarily result in bias [46], we cannot exclude that the patients in our study comprise an a-select sample. A second limitation concerns the comparability of Dutch and Chinese patients. Although patients were similar in terms of gender, and were stratified based on TBI severity and age, we cannot exclude that the patient groups differed on demographic and clinical variables (e.g., education, Glasgow Coma Scale) that were not measured in the Chinese dataset. Related, comorbidity was not assessed in both cohorts, while it is common in TBI patients [47, 48] and could also influence HRQoL [49]. Moreover, the Dutch study administered the SF-36 by a postal questionnaire while the Chinese study used telephone interviews, which might not be comparable. For example, in a telephone interview, social desirability bias is relatively likely to occur [50, 51], which might have resulted in more optimistic results among Chinese patients. Also, a postal questionnaire, especially in patients with severe TBI, might not be reliable because of memory and concentration problems experienced by these patients [52]. Comparability of Dutch and Chinese patients is further hampered by differences in study design; the Dutch database was a prospective cohort study whereas the Chinese dataset was retrospectively collected.

The time between injury and follow-up can also be considered a limitation in this study. Although it is known that a subset of mild and moderate TBI patients experience long-lasting symptoms [44, 49, 53], the majority is expected to be recovered 1 year post-injury [54]. This might have caused the strong ceiling effects in our study. Ceiling effects are considered to be present if the highest score on a subscale is obtained in more than 15% of the respondents [55, 56], which was the case in the majority of subscales for Dutch and Chinese mild and moderate patients. Ceiling effects may reduce reliability and validity of subscales [56] and might indicate that the SF-36 lacks sensitivity to examine differences in TBI patient groups 1 year after the injury. In addition, the skewed distribution might have influenced the validity of the CFA analyses because normality is one of the assumptions of the maximum likelihood method. However, in small sample sizes (N < 200) the maximum likelihood method outperformed other analytic methods such as diagonally weighted least squares [57].

Given these limitations, the findings of current study should be interpreted as preliminary and hypothesis generating. We therefore recommend future studies to use highly comparable patient groups in terms of demographics and clinical variables and a detailed registration of the acute and rehabilitative care provided. Additionally, the inclusion of more objective outcome measurements (e.g., Glasgow Outcome Scale Extended) might provide insight on whether Western and Asian patients experience other symptoms or interpret/cope differently with similar symptoms following injury. Related, next to the SF-36, which is a measurement of general HRQoL, a disease-specific measurement such as the QOLIBRI [58] is recommended to measure the full impact of TBI on HRQoL [59]. In addition, qualitative studies, such as interviews or focus groups might also be suitable to study cultural differences in HRQoL after injury.

Our finding that Chinese mild and moderate TBI patients conceptualize some of the subscales differently, poses a challenge for multi-national trials with HRQoL as outcome measurement. A prerequisite in multi-national trials measuring health status is that the same underlying dimensions are measured and that these dimensions are culturally meaningful in all participating countries [13]. Our research shows that this can be doubted in a TBI population, which was in line with findings in the general population [9, 11]. We therefore recommend multi-national trials including both Asian and Western countries to be cautious in their interpretation of health outcome.

## Conclusions

One year after TBI, Dutch and Chinese patients reported a different pattern of HRQoL. Further, we found cultural differences in the conceptualization of some of the SF-36 subscales, which has implication for outcome evaluation in multi-national trials.

## Additional files

Additional file 1: Hypothesized confirmatory factor analysis. (PDF 9 kb)

Additional file 2: Comparison of Short Form (SF)-36 scales among Dutch and Chinese mild and moderate traumatic brain injury patients for the total population and stratified across three age groups. Description of data: a table that shows the *p*-values of the comparison between Dutch and Chinese mild and moderate traumatic brain injury. We first show a *p*-value in the total population and after that, we divide the population into three age groups and show the *p*-values accordingly. (DOCX 21 kb)

Additional file 3: Item Characteristics of the Short Form (SF)-36 among Dutch and Chinese mild and moderate traumatic brain injury patients. Description of data: The file contains of two tables (one for the Duch patients and one for the Chinese patients) with item characteristics. We show the mean and standard deviation of all SF-36 items, the corrected item-to-scale correlation and the correlation between the item and all other SF-36 subscales. (DOCX 27 kb)

## Abbreviations

AISH: Abbreviated Injury Scale - Head
BP: Bodily pain
ED: Emergency department
GCS: Glasgow Coma Scale
GH: General health
HRQoL: Health related quality of life
ICD: International Classification of Diseases and Related Health Problems
MH: Mental health
PF: Physical functioning
RE: Role limitations related to emotional health problems
RP: Role limitations related to physical health problems
RUBICS: Radboud University Brain Injury Cohort Study
SF: Social functioning
SF-36: Short form 36
SPSS: Statistical package for the social sciences
STROBE: Strengthening the Reporting of Observational Based Studies
TBI: Traumatic brain injury
VT: Vitality
