# Supplementary figures and images for: Comparing health-related quality of life of Dutch and Chinese patients with traumatic brain injury: do cultural differences play a role?
Source: Health Qual Life Outcomes. 2017 Apr 14;15:72. doi: 10.1186/s12955-017-0641-9 (PMC5391570; doi:10.1186/s12955-017-0641-9)

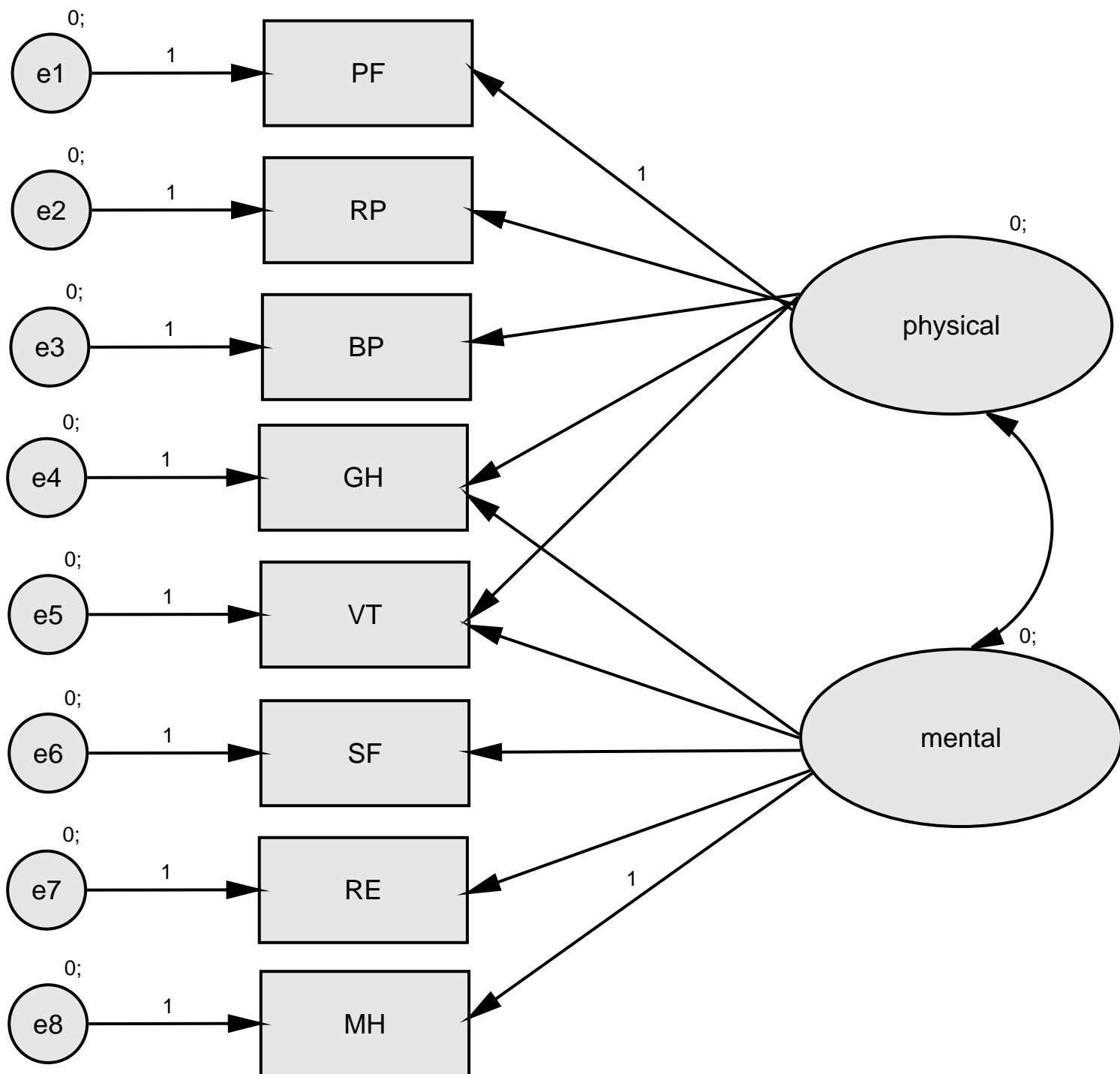

Supplement: Supplementary file 1 — Hypothesized confirmatory factor analysis. (PDF 9 kb) [file 12955_2017_641_MOESM1_ESM.pdf]
