# Supplementary material for: Comparing health-related quality of life of Dutch and Chinese patients with traumatic brain injury: do cultural differences play a role?
Source: Health Qual Life Outcomes. 2017 Apr 14;15:72. doi: 10.1186/s12955-017-0641-9 (PMC5391570; doi:10.1186/s12955-017-0641-9)
Supplement: Supplementary file 2 — Comparison of Short Form (SF)-36 scales among Dutch and Chinese mild and moderate traumatic brain injury patients for the total population and stratified across three age groups. Description of data: a table that shows the p-values of the comparison between Dutch and Chinese mild and moderate traumatic brain injury. We first show a p-value in the total population and after that, we divide the population into three age groups and show the p-values accordingly. (DOCX 21 kb) [file 12955_2017_641_MOESM2_ESM.docx]

**Additional file 2. Comparison of Short Form (SF)-36 scales among Dutch and Chinese mild and moderate traumatic brain injury patients for the total population and stratified across three age groups.**

| **SF-36 scale** | **Total population (N = 377)** | | **Age: 16-31y (n = 120)** | | **Age: 32-49y (n = 118)** | | **Age > 49y (N = 139)** | |
| --- | --- | --- | --- | --- | --- | --- | --- | --- |
|  | **Mean (SD)** | **p-value** | **Mean (SD)** | **p-value** | **Mean (SD)** | **p-value** | **Mean (SD)** | **p-value** |
| PF | 86.3 (22.3) | <.001† | 93.8 (13.1) | .002‡ | 87.8 (22.2) | .001† | 77.6 (26.3) | .041‡ |
| RP | 68.4 (40.6) | .879 | 77.3 (34.1) | .930 | 67.8 (42.9) | .566 | 60.6 (42.5) | .595 |
| BP | 77.1 (26.5) | .001† | 83.4 (22.4) | <.001† | 78.2 (27.3) | .247 | 70.6 (27.9) | .908 |
| GH | 64.1 (23.8) | <.001† | 68.2 (21.7) | .172 | 65.0 (24.3) | <.001† | 59.5 (24.0) | <.001† |
| VT | 65.6 (22.4) | .315 | 66.6 (22.1) | .034‡ | 64.5 (24.3) | .932 | 65.7 (21.0) | .612 |
| SF | 82.9 (22.0) | .026‡ | 85.8 (20.4) | .012‡ | 83.4 (22.6) | .885 | 79.9 (22.6) | .966 |
| RE | 70.6 (33.3) | <.001† | 68.1 (40.6) | .009‡ | 69.5 (38.8) | <.001‡ | 73.8 (38.8) | <.001† |
| MH | 74.9 (20.2) | .601 | 74.3 (20.1) | .566 | 75.5 (21.2) | .632 | 75.0 (19.6) | .517 |

*Note.* Table shows results (p-values) of the Mann-Whitney *U* test comparing SF-36 subscale scores for Dutch and Chinese patients.

† Statistically significant with the stringent criterion (p<.0065)

‡ Not statistically significant with the stringent criteria (p<.0065) but p value below 0.05.

*Abbreviations.* PF = physical functioning; RP = role physical; BP = bodily pain; GH = general health; VT = vitality; SF = social functioning; RE = role-emotional; MH = mental health
