# Supplementary material for: Comparing health-related quality of life of Dutch and Chinese patients with traumatic brain injury: do cultural differences play a role?
Source: Health Qual Life Outcomes. 2017 Apr 14;15:72. doi: 10.1186/s12955-017-0641-9 (PMC5391570; doi:10.1186/s12955-017-0641-9)
Supplement: Supplementary file 3 — Item Characteristics of the Short Form (SF)-36 among Dutch and Chinese mild and moderate traumatic brain injury patients. Description of data: The file contains of two tables (one for the Duch patients and one for the Chinese patients) with item characteristics. We show the mean and standard deviation of all SF-36 items, the corrected item-to-scale correlation and the correlation between the item and all other SF-36 subscales. (DOCX 27 kb) [file 12955_2017_641_MOESM3_ESM.docx]

**Additional file 3. Item Characteristics of the Short Form (SF)-36 among Dutch and Chinese mild and moderate traumatic brain injury patients**

**2A. Dutch data**

| **Item** | **N** | **Mean (SD)** | **Corrected item-to-scale correlation** | **Correlation item and other subscales** | | | | | | | |
| --- | --- | --- | --- | --- | --- | --- | --- | --- | --- | --- | --- |
|  |  |  |  | **PF** | **RP** | **BP** | **GH** | **VT** | **SF** | **RE** | **MH** |
| PF1 | 200 | 2.14 (0.82) | 0.73 | - | 0.61 | 0.56 | 0.51 | 0.39 | 0.44 | 0.32 | 0.33 |
| PF2 | 200 | 2.58 (0.65) | 0.85 | - | 0.73 | 0.62 | 0.56 | 0.44 | 0.60 | 0.45 | 0.36 |
| PF3 | 200 | 2.60 (0.61) | 0.79 | - | 0.68 | 0.58 | 0.44 | 0.34 | 0.49 | 0.33 | 0.30 |
| PF4 | 200 | 2.60 (0.63) | 0.82 | - | 0.57 | 0.47 | 0.45 | 0.34 | 0.43 | 0.27 | 0.28 |
| PF5 | 200 | 2.73 (0.55) | 0.79 | - | 0.47 | 0.43 | 0.33 | 0.20 | 0.36 | 0.27 | 0.21 |
| PF6 | 200 | 2.55 (0.66) | 0.78 | - | 0.57 | 0.57 | 0.47 | 0.30 | 0.45 | 0.30 | 0.26 |
| PF7 | 200 | 2.58 (0.71) | 0.85 | - | 0.64 | 0.52 | 0.51 | 0.39 | 0.51 | 0.34 | 0.34 |
| PF8 | 200 | 2.73 (0.57) | 0.84 | - | 0.56 | 0.45 | 0.46 | 0.32 | 0.47 | 0.35 | 0.29 |
| PF9 | 200 | 2.85 (0.42) | 0.72 | - | 0.41 | 0.31 | 0.29 | 0.20 | 0.30 | 0.26 | 0.20 |
| PF10 | 200 | 2.90 (0.33) | 0.57 | - | 0.38 | 0.33 | 0.25 | 0.11 | 0.26 | 0.24 | 0.07 |
| RP1 | 211 | 0.27 (0.44) | 0.76 | 0.58 | - | 0.55 | 0.55 | 0.51 | 0.60 | 0.54 | 0.41 |
| RP2 | 211 | 0.33 (0.47) | 0.76 | 0.65 | - | 0.57 | 0.60 | 0.52 | 0.58 | 0.57 | 0.47 |
| RP3 | 211 | 0.33 (0.47) | 0.75 | 0.68 | - | 0.64 | 0.56 | 0.43 | 0.54 | 0.45 | 0.40 |
| RP4 | 211 | 0.35 (0.48) | 0.81 | 0.63 | - | 0.68 | 0.61 | 0.51 | 0.61 | 0.49 | 0.43 |
| BP1 | 217 | 2.32 (1.34) | 0.81 | 0.58 | 0.65 | - | 0.52 | 0.42 | 0.49 | 0.36 | 0.33 |
| BP2 | 217 | 1.81 (1.06) | 0.81 | 0.64 | 0.70 | - | 0.56 | 0.44 | 0.54 | 0.36 | 0.35 |
| GH1 | 213 | 3.20 (0.91) | 0.60 | 0.57 | 0.59 | 0.52 | - | 0.57 | 0.54 | 0.38 | 0.46 |
| GH2 | 213 | 4.18 (1.18) | 0.57 | 0.28 | 0.41 | 0.38 | - | 0.51 | 0.47 | 0.32 | 0.51 |
| GH3 | 213 | 3.70 (1.29) | 0.63 | 0.48 | 0.51 | 0.45 | - | 0.46 | 0.45 | 0.26 | 0.42 |
| GH4 | 213 | 3.68 (1.21) | 0.53 | 0.32 | 0.41 | 0.34 | - | 0.39 | 0.41 | 0.29 | 0.39 |
| GH5 | 213 | 3.67 (1.25) | 0.81 | 0.56 | 0.70 | 0.56 | - | 0.65 | 0.63 | 0.52 | 0.58 |
| VT1 | 216 | 2.67 (1.45) | 0.49 | 0.28 | 0.33 | 0.28 | 0.41 | - | 0.44 | 0.38 | 0.55 |
| VT2 | 216 | 2.94 (1.38) | 0.70 | 0.34 | 0.53 | 0.43 | 0.59 | - | 0.58 | 0.49 | 0.44 |
| VT3 | 216 | 2.13 (1.18) | 0.60 | 0.35 | 0.46 | 0.46 | 0.56 | - | 0.59 | 0.49 | 0.41 |
| VT4 | 216 | 3.00 (1.27) | 0.65 | 0.34 | 0.48 | 0.47 | 0.53 | - | 0.60 | 0.44 | 0.57 |
| SF1 | 217 | 1.55 (0.94) | 0.73 | 0.53 | 0.60 | 0.47 | 0.53 | 0.34 | - | 0.64 | 0.65 |
| SF2 | 217 | 2.00 (1.13) | 0.73 | 0.51 | 0.65 | 0.52 | 0.58 | 0.32 | - | 0.62 | 0.66 |
| RE1 | 214 | 0.16 (0.37) | 0.72 | 0.35 | 0.52 | 0.35 | 0.41 | 0.25 | 0.60 | - | 0.57 |
| RE2 | 214 | 0.22 (0.42) | 0.75 | 0.36 | 0.55 | 0.38 | 0.47 | 0.32 | 0.65 | - | 0.65 |
| RE3 | 214 | 0.17 (0.38) | 0.73 | 0.33 | 0.49 | 0.36 | 0.42 | 0.26 | 0.57 | - | 0.55 |
| MH1 | 216 | 2.76 (1.36) | 0.72 | 0.28 | 0.37 | 0.33 | 0.43 | 0.19 | 0.48 | 0.48 | - |
| MH2 | 216 | 2.56 (1.33) | 0.78 | 0.32 | 0.47 | 0.35 | 0.56 | 0.51 | 0.59 | 0.55 | - |
| MH3 | 216 | 2.20 (1.08) | 0.62 | 0.29 | 0.44 | 0.37 | 0.49 | 0.49 | 0.57 | 0.52 | - |
| MH4 | 216 | 1.74 (1.08) | 0.79 | 0.31 | 0.42 | 0.39 | 0.48 | 0.28 | 0.63 | 0.63 | - |
| MH5 | 216 | 2.13 (119) | 0.78 | 0.31 | 0.43 | 0.37 | 0.49 | 0.28 | 0.65 | 0.63 | - |

*Note.* Table presents number of patients that completed the item, mean (SD) of the item, the correlation between the item and all other items of the subscale (“corrected item-to-scale correlation) and the correlation between the item and all other subscales

All items were conversed so that a higher score indicates a better HRQoL.

Abbreviations. PF = physical functioning; RP = role physical; BP = bodily pain; GH = general health; VT = vitality; SF = social functioning; RE = role-emotional; MH = mental health

**2B. Chinese data**

| **Item** | **N** | **Mean (SD)** | **Corrected item-to-scale correlation** | **Correlation item and other subscales** | | | | | | | |
| --- | --- | --- | --- | --- | --- | --- | --- | --- | --- | --- | --- |
|  |  |  |  | **PF** | **RP** | **BP** | **GH** | **VT** | **SF** | **RE** | **MH** |
| PF1 | 153 | 2.61 (0.65) | 0.65 | - | 0.55 | 0.52 | 0.51 | 0.27 | 0.52 | 0.28 | 0.28 |
| PF2 | 153 | 2.89 (0.40) | 0.79 | - | 0.40 | 0.30 | 0.34 | 0.27 | 0.46 | 0.20 | 0.25 |
| PF3 | 153 | 2.88 (0.43) | 0.79 | - | 0.43 | 0.31 | 0.31 | 0.20 | 0.41 | 0.25 | 0.20 |
| PF4 | 153 | 2.80 (0.53) | 0.85 | - | 0.52 | 0.45 | 0.42 | 0.32 | 0.52 | 0.26 | 0.34 |
| PF5 | 153 | 2.90 (0.40) | 0.85 | - | 0.42 | 0.29 | 0.34 | 0.24 | 0.41 | 0.17 | 0.25 |
| PF6 | 153 | 2.81 (0.47) | 0.66 | - | 0.47 | 0.45 | 0.40 | 0.21 | 0.44 | 0.19 | 0.23 |
| PF7 | 153 | 2.89 (0.39) | 0.80 | - | 0.41 | 0.42 | 0.34 | 0.25 | 0.46 | 0.25 | 0.19 |
| PF8 | 153 | 2.95 (0.30) | 0.78 | - | 0.30 | 0.28 | 0.22 | 0.15 | 0.31 | 0.09 | 0.07 |
| PF9 | 153 | 2.95 (0.29) | 0.74 | - | 0.27 | 0.26 | 0.21 | 0.14 | 0.30 | 0.07 | 0.07 |
| PF10 | 153 | 2.94 (0.29) | 0.68 | - | 0.32 | 0.23 | 0.29 | 0.26 | 0.33 | 0.17 | 0.26 |
| RP1 | 153 | 1.72 (0.45) | 0.70 | 0.44 | - | 0.57 | 0.56 | 0.38 | 0.52 | 0.44 | 0.44 |
| RP2 | 153 | 1.69 (0.46) | 0.84 | 0.46 | - | 0.64 | 0.65 | 0.52 | 0.59 | 0.53 | 0.52 |
| RP3 | 153 | 1.67 (0.47) | 0.73 | 0.48 | - | 0.53 | 0.56 | 0.39 | 0.55 | 0.46 | 0.30 |
| RP4 | 153 | 1.67 (0.47) | 0.82 | 0.48 | - | 0.62 | 0.63 | 0.45 | 0.59 | 0.52 | 0.39 |
| BP1 | 153 | 4.93 (1.45) | 0.87 | 0.42 | 0.63 | - | 0.56 | 0.41 | 0.54 | 0.37 | 0.35 |
| BP2 | 153 | 4.40 (1.02) | 0.87 | 0.49 | 0.69 | - | 0.63 | 0.42 | 0.64 | 0.43 | 0.43 |
| GH1 | 153 | 2.95 (1.13) | 0.61 | 0.36 | 0.56 | 0.51 | - | 0.42 | 0.51 | 0.42 | 0.37 |
| GH2 | 153 | 3.18 (1.45) | 0.47 | 0.32 | 0.43 | 0.42 | - | 0.30 | 0.44 | 0.30 | 0.25 |
| GH3 | 153 | 3.67 (1.33) | 0.61 | 0.35 | 0.52 | 0.45 | - | 0.40 | 0.37 | 0.37 | 0.35 |
| GH4 | 153 | 3.54 (1.40) | 0.40 | 0.21 | 0.39 | 0.42 | - | 0.25 | 0.39 | 0.26 | 0.30 |
| GH5 | 153 | 3.26 (1.35) | 0.60 | 0.37 | 0.59 | 0.55 | - | 0.47 | 0.42 | 0.41 | 0.43 |
| VT1 | 153 | 4.33 (1.65) | 0.44 | 0.28 | 0.43 | 0.34 | 0.24 | - | 0.35 | 0.41 | 0.48 |
| VT2 | 153 | 3.53 (1.84) | 0.38 | 0.27 | 0.46 | 0.35 | 0.53 | - | 0.40 | 0.39 | 0.43 |
| VT3 | 153 | 4.93 (1.61) | 0.49 | 0.13 | 0.27 | 0.30 | 0.23 | - | 0.30 | 0.25 | 0.46 |
| VT4 | 153 | 4.52 (1.66) | 0.48 | 0.16 | 0.24 | 0.28 | 0.18 | - | 0.36 | 0.28 | 0.41 |
| SF1 | 153 | 4.45 (0.90) | 0.36 | 0.28 | 0.52 | 0.55 | 0.50 | 0.42 | - | 0.50 | 0.47 |
| SF2 | 153 | 5.19 (1.44) | 0.36 | 0.55 | 0.55 | 0.52 | 0.48 | 0.42 | - | 0.29 | 0.40 |
| RE1 | 153 | 1.65 (0.48) | 0.56 | 0.21 | 0.43 | 0.36 | 0.34 | 0.32 | 0.35 | - | 0.34 |
| RE2 | 153 | 1.56 (0.50) | 0.73 | 0.24 | 0.55 | 0.41 | 0.47 | 0.45 | 0.44 | - | 0.43 |
| RE3 | 153 | 1.45 (0.50) | 0.58 | 0.20 | 0.42 | 0.37 | 0.41 | 0.41 | 0.34 | - | 0.40 |
| MH1 | 153 | 5.20 (1.38) | 0.43 | 0.07 | 0.26 | 0.21 | 0.24 | 0.31 | 0.30 | 0.22 | - |
| MH2 | 153 | 5.29 (1.37) | 0.55 | 0.28 | 0.35 | 0.36 | 0.42 | 0.54 | 0.42 | 0.38 | - |
| MH3 | 153 | 4.35 (1.81) | 0.30 | 0.12 | 0.19 | 0.13 | 0.11 | 0.29 | 0.18 | 0.17 | - |
| MH4 | 153 | 5.20 (1.15) | 0.61 | 0.30 | 0.43 | 0.45 | 0.46 | 0.49 | 0.53 | 0.45 | - |
| MH5 | 153 | 3.84 (1.75) | 0.48 | 0.22 | 0.42 | 0.34 | 0.43 | 0.54 | 0.38 | 0.42 | - |

*Note.* Table presents number of patients that completed the item, mean (SD) of the item, the correlation between the item and all other items of the subscale (“corrected item-to-scale correlation) and the correlation between the item and all other subscales

All items were conversed so that a higher score indicates a better HRQoL.

Abbreviations. PF = physical functioning; RP = role physical; BP = bodily pain; GH = general health; VT = vitality; SF = social functioning; RE = role-emotional; MH = mental health
